# Commentary: Efficacy and safety of magnetic resonance-guided focused ultrasound for Parkinson's disease: a systematic review and meta-analysis

**DOI:** 10.3389/fneur.2026.1798601

**Published:** 2026-04-30

**Authors:** Mostafa A. Khalifa

**Affiliations:** Faculty of Medicine, Cairo University, Cairo, Egypt

**Keywords:** efficacy, magnetic resonance-guided focused ultrasound surgery (MRgFUS), Parkinson, safety, SRMA

## Introduction

Magnetic resonance-guided focused ultrasound (MRgFUS) has emerged as a minimally invasive neurosurgical option for selected patients with Parkinson's disease. In a recent systematic review and meta-analysis, Tian et al. (1) aimed to evaluate the efficacy and safety of MRgFUS by summarizing post-intervention motor outcomes, primarily using MDS-UPDRSIII and CRST scores across 20 studies involving 258 patients. While the article provides a comprehensive synthesis of available data, several methodological aspects warrant further clarification, as they may influence the interpretation of the reported findings.

## Use of absolute post-treatment scores

The meta-analysis was conducted as a single-arm analysis, pooling post-intervention motor scores without incorporating control groups or consistently utilizing baseline-adjusted comparisons. Although such an approach may be appropriate for describing post-treatment outcomes, it does not allow for causal inference regarding treatment effectiveness. Importantly, despite the availability of baseline motor scores across most included studies, the analysis predominantly focused on post-intervention raw mean values rather than within-group change scores or standardized effect sizes. Given the substantial heterogeneity in baseline disease severity across studies (with several analyses demonstrating high *I*^2^ values), this methodological choice may distort the estimated treatment effect.

Baseline-adjusted or change-score-based approaches are methodologically more robust in this context, as they account for between-study variability in baseline severity and provide a more accurate estimate of treatment-related improvement. In contrast, reliance on post-intervention values alone may overestimate treatment benefit, particularly when baseline scores differ substantially across studies, as observed in the included dataset.

## Inconsistency between reported and applied statistical methods

An additional concern is the inconsistency between the statistical methods described and those applied in the analysis. The Methods section specifies that outcomes would be analyzed using mean differences (MD ± SD). However, the results predominantly present pooled post-intervention mean values, and in at least one instance, standardized mean differences are reported without clear methodological justification or consistency across analyses.

This inconsistency introduces ambiguity regarding the analytical framework and raises concerns about the transparency and reproducibility of the findings. Without clear alignment between the stated and implemented statistical methods, it becomes difficult to interpret how effect estimates were derived and to assess the validity of the reported results.

## Discussion

Taken together, these methodological considerations suggest that the findings of the meta-analysis should be interpreted with caution. The results are more appropriately considered descriptive summaries of post-intervention motor outcomes rather than confirmatory evidence of treatment efficacy. This distinction is particularly important in single-arm meta-analyses with substantial heterogeneity, where failure to account for baseline variability may lead to overinterpretation of treatment effects. Clarifying the analytical strategy and, where feasible, re-analyzing outcomes using baseline-adjusted or standardized effect measures would enhance the interpretability and scientific rigor of the study.
